# Restorative proctocolectomy with ileal pouch‐anal anastomosis in elderly patients – is advanced age a contraindication?

**DOI:** 10.1111/ans.17728

**Published:** 2022-04-18

**Authors:** Leonardo C. Duraes, Jennifer Liang, Scott R. Steele, Bora Cengiz, Conor P. Delaney, Stefan D. Holubar, Emre Gorgun

**Affiliations:** ^1^ Department of Colorectal Surgery Cleveland Clinic Foundation Cleveland Ohio USA

**Keywords:** elderly, inflammatory bowel disease, IPAA, ulcerative colitis

## Abstract

**Aim:**

We aimed to determine pouch function and retention rate for restorative proctocolectomy with ileal pouch‐anal anastomosis (IPAA) for ulcerative colitis (UC) in elderly patients.

**Methods:**

We identified patients over 50 years old subjected to IPAA for confirmed pathological UC from 1980 until 2016. Patients were grouped according to age: 50–59, 60–69 and 70+ years. Short and long‐term outcomes and quality of life (QOL) were compared among the groups.

**Results:**

Six hundred and one patients were identified (399 (66.4%) between 50–59 181 (30.1%) between 60–69, and 21 (3.5%) over 70 years of age). More males were in the 70+ arm, and more two‐stage procedures were performed in this group. Wound infection increased with age (*P* = 0.023). There was a trend of more fistula and pouchitis in the 70+ patients (*P* = 0.052 and *P* = 0.055, respectively). Pouch failure rate increased with age, and it was statistically significant in the 70+ cohort (*P* = 0.015). Multivariate stepwise logistic regression showed that pelvic sepsis (HR 4.8 (95% CI 1.5–15.4), *P* = 0.009), fistula (HR 6.0 (95% CI 1.7–21.5), and mucosectomy with handsewn anastomosis (HR 4.5 (95% CI 1.4–14.7)), were independently associated with pouch failure. No difference was observed in the QOL among the groups, but pouch function was better for patients younger than 60 years.

**Conclusion:**

In elderly patients with UC, IPAA may be offered with reasonable functional outcomes, and ileal pouch retention rates, as an alternative to the permanent stoma. Stapled anastomosis increases the chance of pouch retention and should be recommended as long as the distal rectum does not carry dysplasia.

## Graphical abstract

Ileal pouch‐anal anastomosis (IPAA) can be safely performed in patients with ulcerative colitis (UC) over 50 years old, with good outcomes and quality of life (QOL). However, pouch retention rates decrease for patients over 70 years. Stapled anastomosis increases the chance of pouch retention and should be recommended as long as the distal rectum does not carry dysplasia.

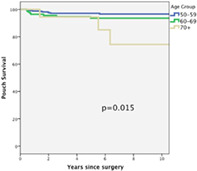

## Introduction

Ileal pouch‐anal anastomosis (IPAA) remains the preferred surgical treatment for the majority of ulcerative colitis (UC) patients.^1^ Though most UC patients are diagnosed during younger ages, due to the bimodal age distribution of UC and recent advancements in medical treatment, the prevalence of the UC in the elderly population is likely to increase.^2^, ^3^ While surgical outcomes of IPAA have been widely studied for younger patients, little is known regarding the long‐term results in respect of de‐novo pouch performed in elderly patients, over 50 years‐old, with presumed weaker sphincter functions.^4^ Indeed, elderly patients are more likely to have comorbidities; therefore, the surgical outcomes of the IPAA surgery may be different in this group of patients.^3^

IPAA has been considered suitable for most patient groups, and there are no exclusion criteria targeted to the patient's age.^5^ Some reports indicate increased postoperative complication rates in elderly patients, but the individual characteristics of the complications and quality of life features are yet to be ravelled.^4^, ^6^, ^7^, ^8^ In this study, we aimed to compare postoperative complications, quality of life, pouch function, and survival in patients older than 50 years and who had undergone de‐novo IPAA surgeries. We hypothesized that the pouch function would be worse in older patients.

## Methods

After Institutional Review Board approval, a prospectively collected pouch database was queried to identify patients older than 50 years of age subjected to IPAA from 1980 until 2016 at the Department of Colorectal Surgery at Cleveland Clinic, OH.

The database variables included: patient demographics, body mass index (BMI), American Society of Anesthesiologists (ASA) classification of physical health, preoperative clinical and pathological diagnosis, details of the surgical procedures, length of hospital stay, perioperative and postoperative morbidity, long‐term outcomes, and quality of life after IPAA surgery.

Only patients with ulcerative colitis at final pathology after colonic extraction were included. Patients with a preoperative diagnosis of UC based on colonoscopy biopsies but who had Indeterminate Colitis or Crohn's Disease in the final specimen were excluded to eliminate confounders. In addition, trans‐abdominal salvage redo/revisionary pouch surgeries were excluded.

Patients were then grouped based on age: 50–59 years, 60–69 years and 70+.

All patients underwent the same standardized IPAA surgery as previously described.^9^ Quality of life was assessed using the Cleveland Clinic Global Quality of Life questionnaire (CGQL).^10^ This questionnaire scores the current quality of life, quality of health, and energy level from 1 to 10. The final CGQL utility score was obtained by dividing these results by 30. The questionnaire also examines dietary, social, work, and sexual restrictions. The questionnaires were self‐administered during follow‐up visits or via mailings.

### Statistical analysis

Analyses were performed using SPSS version 20. Categorical variables were compared using Fisher exact test or *χ* ^2^ test. Comparisons of quantitative and ordinal variables were performed with the Kruskal‐Wallis test. Kaplan–Meier curves were used to display pouch failure percentages across the follow‐up period. Multivariate models were built using a stepwise selection of variables that were significant in the univariate model. A p‐value <0.05 was considered statistically significant.

## Results

From 1980 until 2016, 2855 patients with final pathology of UC were subjected to IPAA at our institution. Of these, 601 patients were over 50 years old (399 patients (66.4%) between 50–59 years old, 181 patients (30.1%) between 60–69 years old, and 21 patients (3.5%) over 70 years old).

There were more males in the 70+ cohort (90.5%) that underwent IPAA compared with other age groups (60–69: 66.9% and 50–59: 64.9%, *P* = 0.04), and more 2‐stage procedures were performed in this group of patients (70+: 85.7%, 60–69: 54.7%, 50–59: 58.4%, *P* = 0.02). No difference was observed in J‐pouch or S‐pouch configuration (*P* = 0.60), stapled versus handsewn anastomosis (*P* = 0.77), laparoscopic or open approach (*P* = 0.92), and diverting ileostomy rates (*P* = 0.87) among the groups. Table 1 summarizes the patient demographics and operative details.

**Table 1:** Demographic and intraoperative details

Group	50–59 (*n* = 399)	60–69 (*n* = 181)	70+ (*n* = 21)	*P*‐value
Age at surgery, years	54.2 ± 2.9	63.4 ± 2.8	73.0 ± 2.3	**<0.001**
Gender, male	259 (64.9%)	121 (66.9%)	19 (90.5%)	**0.043**
Resection type				**0.024**
Total proctocolectomy	233 (58.4%)	99 (54.7%)	18 (85.7%)	
Completion proctectomy	166 (41.6%)	82 (45.3%)	3 (14.3%)	
Pouch configuration				0.602
J‐Pouch	384 (96.2%)	173 (95.6%)	21 (100%)	
S‐Pouch	19 (3.7%)	4 (4.7%)	—	
IPAA anastomosis type				0.776
Stapled	368 (92.2%)	165 (91.2%)	19 (90.5%)	
Hand sewn	31 (7.8%)	16 (8.8%)	2 (9.5%)	
Laparoscopic approach	56 (14.0%)	24 (13.3%)	2 (9.5%)	0.924
Intraoperative diversion	380 (95.2%)	171 (94.5%)	20 (95.2%)	0.879
Dysplasia	65 (16.3%)	34 (18.8%)	5 (23.8%)	0.553
Follow up, years	9.6 ± 7.3	7.5 ± 6.1	7.0 ± 4.6	**0.001**

Data are represented mean ± *SD* or number (percentage) unless otherwise specified. Significant *P*‐values are bolded. IPAA, ileal pouch‐anal anastomosis.

No difference was observed in colorectal dysplasia among age groups (70+: 23.8%, 60–69: 18.8%, 50–59: 16.3%, *P* = 0.553) (Table 1).

Wound infection increased with age (*P* = 0.023). There was a trend of more fistula and pouchitis in patients over 70 years (70+: 14.3%, 60–69: 4.4%, 50–59: 3.3%, *P* = 0.052 and 70+: 38.1%, 60–69: 19.9%, 50–59: 27.8%, *P* = 0.055, respectively) (Table 2).

**Table 2:** Postoperative complications

Group	50–59 (*n* = 399)	60–69 (*n* = 181)	70+ (*n* = 21)	*P*‐value
Anastomotic separation	25 (6.3%)	12 (6.6%)	2 (9.5%)	0.736
Anastomotic stricture	46 (11.5%)	23 (12.7%)	2 (9.5%)	0.913
Fistula	13 (3.3%)	8 (4.4%)	3 (14.3%)	0.052
Haemorrhage	12 (3.0%)	7 (3.9%)	1 (4.8%)	0.538
Obstruction	56 (14.0%)	25 (13.8%)	1 (4.8%)	0.603
Pelvic sepsis/chronic abscess	28 (7.0%)	12 (6.6%)	3 (14.3%)	0.347
Pouch failure	10 (2.5%)	8 (4.4%)	3 (14.3%)	**0.023**
Pouchitis	111 (27.8%)	36 (19.9%)	8 (38.1%)	0.055
Wound Infection	34 (8.5%)	27 (14.9%)	4 (19.0%)	**0.023**
Other	210 (52.6%)	87 (48.1%)	15 (71.4%)	0.113

Data in number/percentage, otherwise specified. Statistically significant *P*‐values are bolded.

**Fig. 1:**
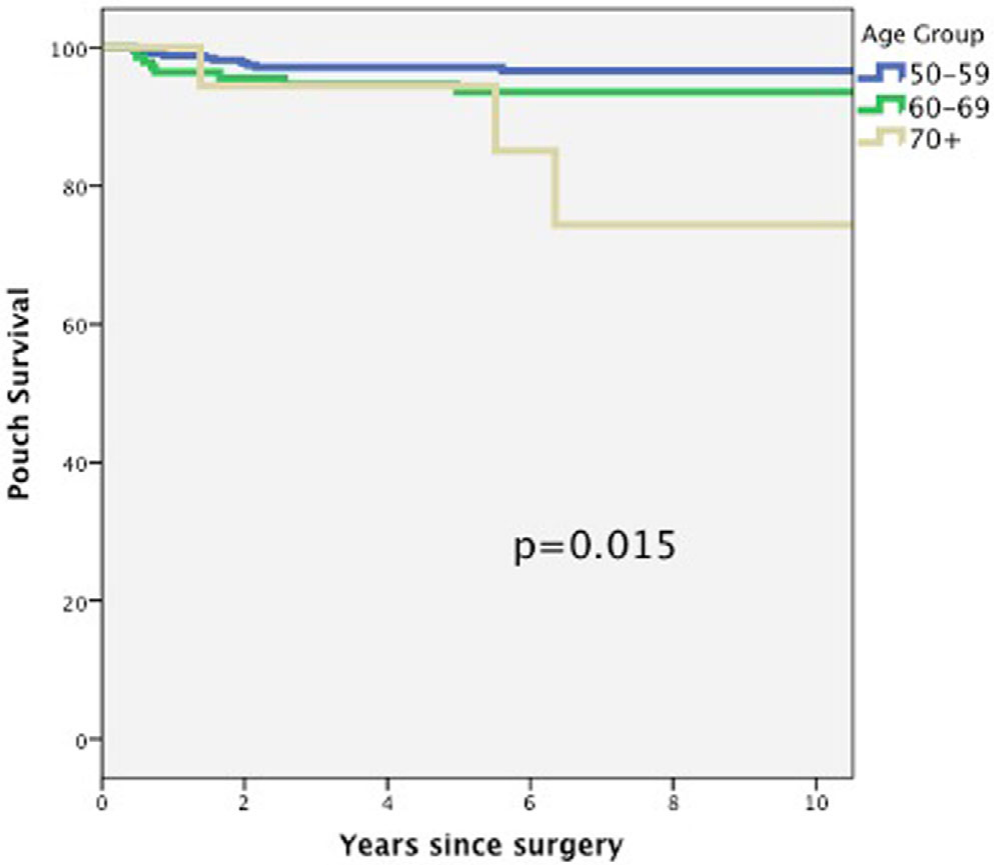
Ileal pouch Kaplan–Meier survival curve by age groups.

Pouch failure rate increased with age, and it was statistically significant in patients older than 70 years‐ old (*P* = 0.015) (Fig. 1) (Table 2). The pouch failure in patients over 70 years occurred at a later stage of follow‐up (median 5.5 years), compared to 50–59 and 60–69 years old groups (1.5 and 0.7 years, respectively). The reasons for pouch failure in patients older than 70 years old were anal canal cancer, faecal incontinence with skin excoriation, and pouch cutaneous fistula.

Multivariate stepwise logistic regression showed that pelvic sepsis (HR 4.8 (95% CI 1.5–15.4), p = 0.009), fistula (HR 6.0 (95% CI 1.7–21.5), *P* = 0.006), and mucosectomy with handsewn anastomosis (HR 4.5 (95% CI 1.4–14.7), *P* = 0.012) were independently associated with pouch failure in the elderly population (Table 3).

**Table 3:** Multivariate stepwise logistic regression for pouch failure

Variable	Hazard Ratio (95% confidence interval)	*P*‐value
Pelvic sepsis	4.8 (1.5–15.4)	**0.009**
Presence of fistula	6.0 (1.7–21.5)	**0.006**
Handsewn anastomosis	4.5 (1.4–14.7)	**0.012**

Significant *P*‐values are bolded.

When we looked at the CGQOL, no difference was observed among the age groups in quality of health (0.453), energy level (*P* = 0.097), quality of life (*P* = 0.097) and overall CGQOL score (*P* = 0.306) (Table 4). However, patients older than 60 years old had more seepage during the day (*P* < 0.001) and during the night (*P* = 0.028) (Table 4).

**Table 4:** Quality of life and functional outcomes

Variable	50–59 (n = 399)	60–69 (*n* = 181)	70 + (*n* = 21)	*P*‐value
Overall CGQOL score (mean, *SD*)	0.73 ± 0.18	0.75 ± 0.16	0.71 ± 0.17	0.306
Quality of health (mean, *SD*)	7.6 ± 1.8	7.6 ± 1.8	7.1 ± 1.9	0.453
Energy level (mean, *SD*)	6.7 ± 2.0	7.1 ± 2.0	6.6 ± 1.8	0.097
Quality of life (mean, SD)	7.6 ± 1.9	7.9 ± 1.6	7.6 ± 1.8	0.480
Dietary restriction	129 (36.6%)	58 (36.0%)	4 (28.6%)	0.886
Social restriction	79 (22.6%)	33 (20.9%)	4 (25.0%)	0.837
Work restriction	67 (19.6%)	29 (19.3%)	3 (20.0%)	1.000
Sexual restriction	115 (33.5%)	42 (28.4%)	3 (21.4%)	0.426
Would undergo surgery again	197 (92.9%)	80 (92.0%)	12 (92.3%)	0.925
Surgery satisfaction	5.5 ± 4.3	5.3 ± 4.3	6.7 ± 3.6	0.527
Would recommend surgery	203 (95.3%)	79 (90.8%)	11 (84.6%)	0.113
Pouchitis symptoms	100 (33.4%)	37 (29.6%)	4 (30.8%)	0.725
Seepage during the day	58 (26.7%)	44 (48.9%)	6 (42.9%)	**<0.001**
Seepage during the night	86 (39.6%)	51 (56.0%)	7 (50.0%)	**0.028**

Data in number/percentage, otherwise specified. Statistically significant *P*‐values are bolded.

## Discussion

The present study demonstrates that IPAA can be safely performed in patients older than 50 years old with acceptable functional outcomes. However, a higher pouch failure rate was found in the 70+ patients compared with 50–59 and 60–69‐year‐old patient groups. Pelvic sepsis, fistula and handsewn anastomosis were the factors independently associated with pouch failure. Therefore, to increase the pouch survival rate, stapled anastomosis should be offered to 70+ patients who are suitable for this technique.

IPAA is the preferred surgical treatment for UC with good outcomes and QOL.^1^, ^9^, ^10^ The IPAA surgery in elderly patients remains controversial.^2^, ^3^, ^6^, ^11^ IPAA remains a major operation, with high morbidity and mortality rates, and these complication rates are increased in older patients.^2^, ^8^, ^12^ Another point to consider is that elderly patients have weaker sphincter function, which can lead to poor pouch function.^4^, ^7^, ^13^, ^14^ Moreover, the elderly population with UC have higher dysplasia rates; therefore, there is a higher risk of cancer in the pouch or anal transition zone.^12^, ^15^ In order to avoid any confusion, we used only patients with surgical pathology consistent with UC, different from previous studies that used preoperative biopsies to select patients.^14^

Although the morbidity rate was high in our study, no difference was observed among the groups in most of the complications. Wound infection was the only complication with a statistical difference, increasing its incidence after 60 years of age. There was a trend in fistula and pouchitis incidence with ageing; however, no statistical difference was found. Therefore, in our study, morbidity was not a factor that would lead us to an age cutting for an IPAA surgery in the elderly patient. Most studies show increased morbidity with ageing, but no studies showed a cutting age for the operation.^2^, ^6^, ^8^, ^11^, ^13^, ^16^

The function of the pouch can be an issue due to the weaker sphincter muscles. Our study shows that patients older than 60 years old have more seepage during the daytime and night. Nevertheless, the quality of life was similar in all groups, despite the higher seepage. Our results go along with the available literature, where the pouch function worsens with ageing, but patients have an acceptable QOL.^4^, ^7^, ^13^, ^14^ Our results are reassuring for surgery in terms of quality of life. Again, no age cut‐off was found for QOL outcomes, but patients older than 60 should be aware of functional consequences, especially the risk of seepage and incontinence.

One of the main concerns on the pouch in the elderly population is dysplasia and potential cancer risk. In our study, dysplasia incidence was similar among the age groups and did not increase the cancer incidence. Our results also go along with literature where IPAA in the elderly population was not associated with cancer.^17^, ^18^ Although 24% of the patients over 70 years old had dysplasia, only one patient who underwent IPAA in this age group had anal transition zone (ATZ) cancer during follow‐up. Mucosectomy is not recommended in this age group due to the risk of pouch failure. Again no age cut‐off for IPAA surgery was identified in terms of dysplasia.

In general, our elderly patient population, over 50 years old, had an excellent pouch retention rate of 96% in a median follow‐up of 8 years. However, patients older than 70 years had a pouch survival rate of 85%. Factors associated with pouch failure were fistula, pelvic sepsis and handsewn anastomosis. Therefore, double‐stapled anastomosis should be strongly recommended in elderly patients. In our cohort, the great majority of the handsewn anastomosis (78%) were performed in the first half period (1980–1998). The numbers of handsewn anastomosis decreased over time. Stapled anastomosis is our current standard of care whenever possible, especially in the elderly population.

Most of the studies available in the literature compared older patients against younger ones. No previous studies were designed to know when it would be too old for the IPAA. Our study shows that the significant difference occurs in patients older than 70 years old, mainly for pouch retention, and older than 60 years for pouch function and morbidity (mainly wound infection).

Our study has limitations, such as retrospective study, single‐centre and possible selection bias for surgery in elderly patients. However, this is big data from a high‐volume institution, with extended follow‐up, which provides us a unique opportunity of identifying outcome predictors.

In conclusion, IPAA may be offered to selected elderly patients with ulcerative colitis, with reasonable functional outcomes, and good ileal pouch retention rates, as an alternative to the permanent stoma. Although, pouch retention rates were lower in patients older than 70 years old in long‐term follow‐up. Stapled anastomosis increases the chance of pouch retention and should be recommended as long as the distal rectum does not carry dysplasia.

## Conflict of interest

None declared.

## Ethics approval

This study has been approved by the Institutional Review Board.

## Author contributions

**Leonardo C. Duraes:** Conceptualization; data curation; formal analysis; methodology; writing – original draft; writing – review and editing. **Jennifer Liang:** Conceptualization; data curation; methodology; writing – review and editing. **Scott R. Steele:** Conceptualization; data curation; methodology; supervision; writing – review and editing. **Bora Cengiz:** Data curation; formal analysis; investigation; visualization; writing – review and editing. **Conor P. Delaney:** Conceptualization; investigation; methodology; supervision; validation; writing – review and editing. **Stefan D. Holubar:** Data curation; investigation; methodology; validation; writing – review and editing. **Emre Gorgun:** Conceptualization; data curation; formal analysis; methodology; supervision; validation; writing – original draft; writing – review and editing.
